# Practicing voice: student voice experiences, democratic school culture and students’ attitudes towards voice

**DOI:** 10.1080/02671522.2023.2178496

**Published:** 2023-03-02

**Authors:** Willemijn F. Rinnooy Kan, Anke Munniksma, Monique Volman, Anne Bert Dijkstra

**Affiliations:** Department of Child Development and Education, University of Amsterdam, Amsterdam, the Netherlands

**Keywords:** democratic attitudes, secondary schools, student voice, democratic school culture, open classroom climate

## Abstract

The abilities of citizens to make themselves heard and listen to each other are essential for the functioning of democratic societies. Schools are practice grounds for these citizenship competences. This study investigates whether students’ experiences with voice in school are related to their attitudes towards voice (contributing and listening democratically), and how a democratic school culture affects this relation. Overall, 5297 students, from 240 classrooms, in 81 Dutch secondary schools, participated in the study. Results of multilevel analyses revealed that students’ voice experiences at school, their own and those of their classmates, are positively related to students’ attitudes towards contributing and listening democratically. This relation is not affected by a democratic school culture. These findings underline the relevance of opportunities to practice voice at school for all students and of the social nature of practicing voice. More generally, this study illustrates the importance of understanding schools as practice grounds for citizenship.

## 1. Introduction

Voice has always been understood as a key aspect of democracy, Aristotle already connected man’s political nature to the capacity to speak and reason (Mulgan 1974). Moreover, democracy itself is defined by rule of the people through their voice:

*In a meaningful democracy, the people’s voice must be loud and clear: clear so that policymakers understand citizen concerns and loud so that they have an incentive to pay attention to what is said*. (Verba, Schlozman, and Brady 1995, 1)

This example illustrates that voice can only serve its purpose when there is someone listening. Therefore, listening can be considered an equally essential aspect of democracy through voice, although it is rarely researched in such a way (Dobson 2014). For the longevity of our democratic system, the way citizens relate to their own voice and that of others matters. This holds especially true for youth: the voters and representatives of the future.

Meanwhile, across the globe, academics in various disciplines have pointed at the lack of democratic attitudes and engagement of adolescents (cf. De Groot and Veugelers 2015). One of the ways to address these supposed deficits is through (citizenship) education (Quintelier 2010). Besides the explicit transfer of knowledge and skills related to citizenship, schools can be understood as practice grounds for adolescents’ citizenship, where students’ practice their citizenship through daily experiences in and outside the classroom. Adolescent citizenship can be understood as a developmental practice related to community membership. Voice and listening are both key aspects of this practice (Lawy and Biesta 2006). Insight into what kind of practice matters for the development of student’s attitudes concerning voice, their own voice and that of others, helps us to understand how educators can fulfil their role as socialising agents in the development of students’ citizenship, and consequently, as crucial contributors to the proper functioning of our democracies.

Students can practice with different kinds of voice in school. Firstly, voice can be defined as ‘active participation in deliberation about decisions and events’ (Thomson 2011, 21). This expression of voice can be referred to as ‘voice as discussion’. Research indicates that in the school context an open classroom climate, which facilitates the opportunity to discuss and debate political, social and controversial topics and to explore different perspectives, plays an important role in the development of citizenship of adolescents (e.g. Alivernini and Manganelli 2011; Barber, Sweetwood, and King 2015; Campbell 2008; Maurissen 2018). Voice, however, can also be used to describe access to those who make decisions, or to partaking in decision-making itself (Thomson 2011). This expression of voice can be referred to as ‘voice as influence’. In schools, this could, for example, mean taking part in the student council or being part of an advisory group for a specific policy decision. A review study on this type of student voice indicated (moderate) positive effects of ‘student involvement in collective decision-making processes at the school’ (Mager and Nowak 2012, 40) on diverse citizenship-related student outcomes (Mager and Nowak 2012).

Furthermore, research indicates that within the school as practice ground there are different mechanisms at play through which students’ experiences at school relate to their citizenship. First, students practice their citizenship through daily experiences at school, such as solving conflicts with their fellow classmates or participating in a debate on current affairs in the classroom (Lawy and Biesta 2006; Rinnooy Kan et al. 2021). These active individual experiences may lead to improved civic skills and consequently to more democratic attitudes (Quintelier and Van Deth 2014). Secondly, social learning theory suggests that students can additionally learn from modelling by their fellow students and teachers, for example when they see their fellow classmates using a recycle bin for their garbage, or their teacher listening attentively (Bandura and Walters 1977). Finally, students learn about citizenship through being part of the broader school culture, which may represent a certain ‘norm’ regarding citizenship-related aspects such as participation, cooperation, and voice, increasing (or diminishing) the effect of their other, more direct, citizenship-related experiences (Mitra, Serriere and Stoicovy 2012; Lenzi et al. 2014; Wood 2014).

We complement the current research by building on the idea of ‘schools as practice grounds for citizenship’ and relating ’voice’ as it is practiced *inside* the school to thematically corresponding citizenship-related learning outcomes; we focus on students’ attitudes towards voice – using one’s own voice and listening to that of others – as democratic practices. By combining the three mechanisms: what is practiced by students, what is modelled by their fellow students and teachers, and the role of the school culture, we aim to get more insight into how schools work as a practice ground for citizenship, and as such contribute to the citizenship of their students. Concluding, our two research questions are as follows: Are students’ experiences with voice in school related to students’ attitudes towards voice? And, does the broader school culture influence this relationship?

## 2. Conceptual framework

### 2.1. Students’ attitudes towards voice

Students’ attitudes towards voice can be considered part of their broader democratic attitudes. Democratic attitudes are attitudes individuals hold towards the functioning of a democratic system and their own role therein. Although these attitudes have been operationalised in different ways in survey research with adolescents, key elements that are often included are as follows: interest in, trust towards the functioning of, and a belief that one can contribute to the democratic system (e.g. Dassonneville et al. 2012; Quintelier and Van Deth 2014; Perliger, Canetti-Nisim, and Pedahzur 2006).

Democratic attitudes are understood to be related to participation in democratic societies, and therefore, supporting students to develop and internalise these attitudes is considered one of the main aims of citizenship education (cf. Catterberg and Moreno 2006; Sullivan and Transue 1999). Furthermore, democratic attitudes seem to stabilise by the end of adolescence, another reason for educators to reflect on how students can best be supported in and invited to develop these attitudes during this period. One of the explanations for this stabilisation is the ‘impressionable years hypothesis’ (Sears and Levy 2003) that stipulates that it is during the years of adolescence and early adulthood that the process of political socialisation has the most profound influence. Although empirical research shows mixed results when testing this hypothesis (e.g. Alwin and Krosnick 1991; Osborne, Sears, and Valentino 2011), the results do indicate that especially in the realm of the formation of democratic attitudes, early adolescence is a crucial phase and attitudes seem to stabilise later in adolescence (from 15 years onwards) (e.g. Eckstein, Noack, and Gniewosz 2012).

More specifically, when it comes to attitudes towards ‘voice’, we can distinguish between attitudes towards one’s own voice and that of others. Accordingly, using one’s voice or contributing democratically needs to be accompanied by listening democratically, engaging with the other side of voice in the light of ‘democratic communication’ (Dreher 2012). The abundant theorising on voice in the context of democracy stands, however, in stark contrast to the minimal reflections on the virtue and skill of listening in the context of democracy (Dobson 2014). This holds especially true for adolescents, where the sparse research considering their attitudes towards voice primarily focusses on their intention to use their own voice in the future by voting in an election (e.g. Cohen and Chaffee 2013). In a recent study, students attributed more importance to voting when they experienced more education around social and civic issues in their schools that were explored through student-led and interactive pedagogies, indicating the importance of the practice of voice for the attitudes towards it (Weinberg 2020).

Notwithstanding the relevance of students’ (attitudes towards) ‘normative political participation’, such as voting, this focus is too narrow and limits our understanding of the ways students actually engage politically and use their voice (Edwards 2009; Manning 2013). A broader range of expressions of voice, beyond the institutional and the formal, has been suggested to be relevant ways in which voice is part of student’s citizenship (cf. Kaulingfreks 2015). This implies that concerning students’ attitudes towards voice, their own voice and that of others, expressions closer to their daily experiences might yield more relevant insights than a focus on intended future use of their voice. In this study, we use the concepts contributing and listening democratically to describe practices that are indeed closer to students’ daily life. ‘Contributing democratically’ thus refers to (attitudes towards) contributing to a more just world, as well as discussing current events and the news. ‘Listening democratically’ refers to (attitudes towards) listening to (different) opinions and ideas of other people.

### 2.2 Schools as practice grounds for citizenship

Adolescents practice and develop their citizenship through social interactions with others (Biesta and Lawy 2006; Lawy and Biesta 2006; Torney-Purta and Amadeo 2011). Schools, in this context, can be understood as practice grounds, where adolescents do not only learn about their future as citizens but practice it with fellow students and teachers (Rinnooy Kan et al. 2021; Veugelers 2011). The idea of schools as practice grounds for citizenship finds its roots in the work of John Dewey (1903), who defined democracy through a broad associationist lens, not only as a concept applicable to the political organisation of a nation but as a concept applicable to the functioning of all communities. In his work, he specifically focused on the school community, and the importance of experiential learning and a democratic school organisation for students to be able to practice living in ‘association’ with each other. Research indicates that practicing with, and experiencing elements of, the functioning of democracy promotes the development of democratic attitudes (see e.g., Quintelier and Van Deth 2014).

In the context of schools as practice grounds, there are three mechanisms at play, as mentioned in the introduction, through which students develop their citizenship. Firstly, students develop their citizenship through individual citizenship learning opportunities, such as taking part in a debate or trying to develop a system to recycle garbage in school. Secondly, as stipulated by social learning theory, they develop their citizenship through the modelling of their peers and teachers (Bandura and Walters 1977). A good example of where this modelling of citizenship takes place is in the context of an ‘open classroom climate’ where teachers invite students to share their ideas and listen to those of others. Research indeed consistently shows the importance of an open classroom climate for students’ citizenship (Barber, Clark, and Torney-Purta 2021; Campbell 2008; Geboers et al. 2013). Thirdly, as Dewey already argued, the broader school culture matters for the development of students’ citizenship. It provides the character of, and norms for, the community in which students practice their citizenship. Hence, the democratic character of the school culture might strengthen the association between individual and social experiences of students and their citizenship. For example, research indicates that when students experience a democratic school culture this positively relates to their civic engagement (Lenzi et al. 2014).

### 2.3. Practicing student voice

A central aspect of citizenship learning opportunities within the school context is ‘voice’. The use of the concept voice stems from the social movements of the sixties and seventies. It was used for those who were not members of the traditional dominant group in society ‘voicing’ their ideas, being heard and taken seriously, but also to describe actual influence on governing processes in terms of representation (Thomson 2011). Voice as such can be defined as an opportunity to express opinions, but also as ‘active participation in deliberation about decisions and events’ (Thomson 2011, 21). In the context of schools, student voice can refer to both these expressions: being heard and voicing ideas within the classroom and being engaged in processes of decision-making within the school. We refer to these as ‘voice as discussion’ and ‘voice as influence’. Furthermore, in line with the previous conceptualisation of students’ attitudes towards voice, practicing voice in both these ways is not only about using your own voice, it also implies practicing with listening and being open to other voices.

**Figure 1.**
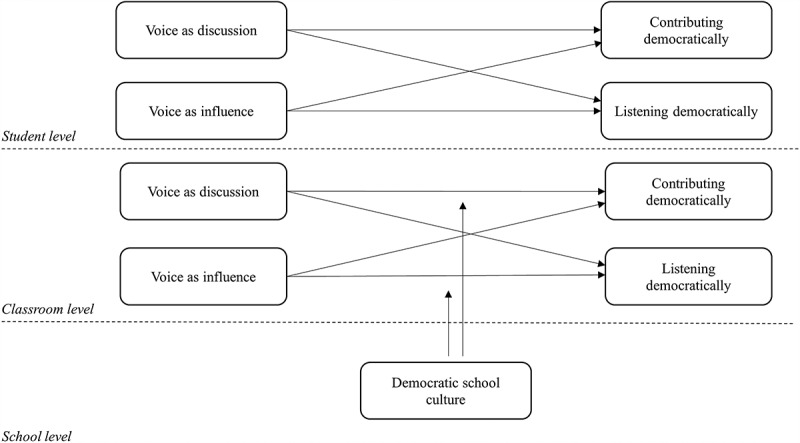
Conceptual model

The opportunity to express one’s opinion and partake in discussion in school is usually researched in the context of an open classroom climate. In this study, we refer to this expression of voice as ‘voice as discussion’. Several studies have pointed out that an open classroom climate for discussion is related to citizenship-related outcomes, such as: civic knowledge (Alivernini and Manganelli 2011; Barber, Sweetwood, and King 2015; Campbell 2008), appreciation for political conflict (Campbell 2008), sociopolitical efficacy and critical action in the community domain (Godfrey and Grayman 2014), political trust (Claes, Hooghe, and Marien 2012) and expected electoral participation (Castillo et al. 2015). In an open classroom climate not only the voice of individual students is invited, but it is also safe to express an opinion or ideas that differ from those of your fellow students or your teacher and consequently in an open classroom climate others are listening. An open classroom climate can be understood as something that is experienced individually (Mechanism 1), but additionally it can also be understood as a characteristic of a classroom (Mechanism 2), which may be established by measuring the experiences of the classroom as a group. In line with the idea of schools as practice grounds for citizenship, we therefore hypothesise that experiences with voice as discussion are related to more positive attitudes on a) contributing democratically, and b) listening democratically (*Hypothesis 1 a* *and b -* see: Figure 1).

The second expression of voice relates to influencing decisions or policies within the school. For students, this experience is primarily available through participation in the student council. In this study, we refer to this expression of voice as ‘voice as influence’. A review of research on student participation in school decision-making (Mager and Nowak 2012) showed that most studies related this type of participation to citizenship outcomes of students and indicated moderate positive effects on civic knowledge, increased understanding of democratic processes and practices and democratic skills and values. Other studies also highlight the importance of experiencing this type of voice and ownership for civic outcomes (Morgan and Streb 2002). Nonetheless, although student councils hold the potential to experience partaking in policy development, the downside is that in general it is only open to a select group of students that can be democratically chosen or simply appointed. Individual experiences of voice on the level of influencing policy could positively contribute to students’ attitudes towards voice (Mechanism 1), but additionally, these experiences could be relevant on the level of the whole class; when students see this type of voice modelled by their fellow students this could influence their perceptions of the possibilities of contributing and listening democratically (Mechanism 2). Based on this, we hypothesise that experiences with voice as influence are related to more positive attitudes on a) contributing democratically, and b) listening democratically (*Hypothesis 2 a and b -* see: Figure 1).

### 2.4. Democratic school culture

When schools are understood as practice grounds for citizenship, it is not only the direct citizenship learning opportunities present for students that are relevant but additionally what is practiced within the school by members of the professional community (mechanism 3) matters. A whole-school approach, including teacher and school leader experiences, has been argued to be crucial to understand how schools contribute to the development of citizenship of adolescents (Barber, Clark, and Torney-Purta 2021; Furman and Starratt 2002; Levinson and Brantmeier 2006). A whole-school approach centres around the idea that students’ learning experiences inside the classroom are influenced and shaped by formal and informal messages ‘promoted by the school’s values’ and the actions of all members of the professional community (Gibb 2016, 3).

In coming to grips with the different ways students’ citizenship is supported in schools, Wood (2014) proposes that schools can be defined by their ‘participatory capital’. This refers to the way participation is invited and practiced within the school and is understood as a characteristic of the functioning of the entire school community, including the members of the professional community that together adhere to a ‘shared habitus of participation’ (Wood 2014, 590). Although the way participation is invited and supported in the professional community might not have a direct effect on students’ attitudes towards voice, through this understanding of ‘shared habitus’ it is exemplary for the way participation is practiced in schools and can be understood as part of the context in which students’ experiences are embedded. A study by Reichert, Chen, and Torney-Purta (2018) showed that in schools where the level of ‘teacher participation in school governance’ was higher, chances of having a student body that used both types of their ‘voice’ (i.e., being an active participant in classroom discussions and/or being active in the student council), were also higher. We therefore hypothesise that positive teacher experiences with voice in school, as part of a democratic school culture, positively moderate the relationship between student voice and students’ democratic attitudes concerning voice (*Hypothesis* *3-* see: Figure 1). We distinguish between a moderation (by teachers’ voice experiences in school) of the relation between:

- voice as discussion and contributing democratically (*Hypothesis 3a*)
- voice as influence and contributing democratically (*Hypothesis 3b*)
- voice as discussion and listening democratically (*Hypothesis 3c)*
- voice as influence and listening democratically (*Hypothesis 3d*).

## 3. Method

### 3.1 Data

Data come from the large-scale research project *Understanding the Effects of Schools on students’ Citizenship*. This project aims to investigate citizenship education and citizenship competences of 9^th^ grade students in secondary schools in the Netherlands. In total, 81 secondary schools, 240 classes and 5,297 students participated in this study. Per school, students from three third year classes (14/15 year-olds), their mentors, 12 other teachers and one principal were invited to participate in the study.

### 3.2 Procedure

Schools were recruited for this study through two different routes. The first route was a random sampling procedure (*n* = 52). To increase statistical power, a second route was employed, using the research team members’ social networks (*n* = 30). After schools agreed to participate, three third-grade classrooms per school were randomly selected. If possible, classrooms of different educational tracks were included. The parents of the students were informed about the study and could deny consent for their children to participate in the study. Students were also informed about the study, including information about confidentiality and that participation was voluntary. Per school class, students filled in two questionnaires in the classroom during two regular classes, under the supervision of a trained test leader. The first questionnaire mainly contained questions about students’ background and their perception of school characteristics. The second contained questions about citizenship competences and citizenship education (Ten Dam et al. 2011).

In addition, 15 teachers were invited to fill in a questionnaire, focusing on school climate and citizenship education. Per school, three of the participating teachers were the mentors of the participating classrooms. Furthermore, schools were given instructions on how to randomly select 12 teachers across all subject areas that taught the participating classrooms. They all received a link to an online questionnaire that they filled in individually. On average nine teachers participated per school.

### 3.3 Analysis sample

Three schools were excluded from the analyses sample because they did not meet the selection criteria of the study. This concerned one school for special needs education, and two schools that shared a location, and interrelatedness of these schools (and their students and teachers) is not clear (see for the procedure Coopmans et al. 2020). Additionally, two classrooms with less than five participating students (i.e., one and four students) were excluded from the current analyses because responses of less than five students do not represent classroom-level outcomes. The resulting analysis sample consists of 78 schools, 230 classrooms, 5167 students, and 643 teachers. The students were on average 14 years old, 51,6% was female. Students were part of classrooms from different educational tracks: lower-level (43,5% VMBO, preparatory secondary vocational education) middle-level (23,2% HAVO, general secondary education), higher-level (25,7% VWO, pre-university education), mixed middle and high (6,7%), and mixed lower and middle/high (0,9%) educational track. Participating teachers were on average 42 years old, working on average 10,64 years in this school, and 53,7% was female. Teacher data is missing for three schools.

### 3.3 Measures

#### 3.3.1. Attitude towards listening democratically

Students indicated to what extent they agreed with the following three statements: (1) people should listen to each other, also when they differ in their opinions; (2) if someone in our class disagrees with something, he/she should have the opportunity to explain why; and (3) in a discussion everyone should have the opportunity to contribute. Students answered on a 4-point Likert-scale, ranging from, 1, *not applicable at all* to (4) *very applicable*. A higher score means these statements suited the student better. The scale was created by taking the mean. These three items formed a reliable scale (Cronbach’s α = .81).

#### 3.3.2. Attitude towards contributing democratically

Students indicated to what extent the following three statements applied to them: (1) I like to discuss what happens in the world with others; (2) when we discuss the news in class, I like to contribute; and (3) I believe it is important that children and adolescents dedicate themselves to a just world. Students answered on a four-point Likert-scale, ranging from, 1, *not applicable at all* to, 4, *very applicable*. A higher score on the scale means these statements were more applicable to the student. The scale was created by taking the mean. The internal consistency of the scale was good (Cronbach’s α = .74).

#### 3.3.3. Voice as influence

Students indicated how often they participated in the following two activities at this school: (1) deciding how things are done at school and (2) being a candidate for class representative or the student council. Students answered on a scale from 1, *never*, to 4, *(very) often*. A dummy was created with 0 for never having undertaken any of the two activities, versus 1 for having undertaken at least once one of the activities. The correlation between these two activities was *r* = .36. Additionally, to use this variable at the classroom level, the percentage of students participating in these activities was computed per classroom.

#### 3.3.4. Voice as discussion

Students indicated on a scale from 1, *(almost) never*, to 5, *(almost) always*, how much they agreed with six statements. Example statements are as follows: ‘students are invited to suggest topics for discussion’ and ‘teachers try to get students to express their own opinions’. These items form a reliable scale (Cronbach’s α = .80). The scale was created by taking the average of all items. A higher score on the scale indicates that students experience a more open climate for discussion at school. To use this variable at the classroom level, the classroom average was taken.

#### 3.3.5. Democratic school culture

Teachers indicated on a scale from 1, *strongly disagre*e, to 5, *strongly agree*, how much they agreed with seven statements. Two example items are as follows: (1) the school leader really listens to teachers who experience problems with new policies and (2) the school leader invites teachers to contribute new ideas and information that fit the school’s profile and to discuss their findings with colleagues. The internal consistency of this scale was high (Cronbach’s α = .90). The scale was created by taking the average of all items. A higher score on the scale indicates a stronger sense of the school climate being democratic. Per school, the average perceived teacher voice was calculated.

#### 3.3.6. Student level control variables

Gender was measured by self-report and coded as zero for boys and one for girls. Socio-Economic Status (SES) was based on the educational level of the parent with the highest educational level. This was dummy coded to 0, *lower SES* (up to intermediate vocational education), and 1, *higher SES* (higher vocational education (HBO) and higher). Migration background was computed based on the countries of birth of both parents. Following Statistics Netherlands (2019), when at least one of the parents was born outside the Netherlands, the student was considered as having a migration background. This was dummy coded as 0, *no migration background*, and 1, *migration background*.

#### 3.3.7. Classroom level control variable

Because Dutch schools are tracked, we control at the classroom level for whether students are in the (0) *vocational track* (vmbo/lower) and the (1) *general/academic educational track* (havo and vwo/higher) at the classroom level. Since there were only two mixed lower plus middle/higher track classrooms, this was not included in the analyses for these classrooms (and the track was there coded as missing).

### 3.4 Analytical Strategy

The analyses proceeded in two steps. First, descriptive statistics were calculated to get insight in the data. Second, the hypotheses were tested using three-level multivariate structural equation models (in Mplus: Muthén and Muthén 1998-2018) to account for the nested structure of the data (students in classrooms within schools) and the interrelatedness of the two dependent variables (contributing democratically and listening democratically). This allowed examining how both individual level and classroom level experiences with voice (as discussion and as influence) are related to the dependent variables. The stepwise procedure was followed to build the model. Democratic school culture was added as a moderator at the school level. All continuous variables were grand mean centred for model estimation. All models showed good model fit (CFI > .95; TLI > .90, RMSEA < .05).

Preliminary analyses indicated that the dependent variables did not follow a normal distribution. Hence, Maximum Likelihood Estimation with Robust Standard Errors (MLR) was used in the multilevel structural equation models. This reduces the bias in standard errors to which non-normal data are prone. Cases with missing data were taken into account with all available data (using FIML in Mplus).

## 4. Results

### 4.1 Descriptive analyses

Table 1 shows descriptive statistics of all main variables in this study. Regarding the dependent variables in this study, preliminary analyses show that students in general endorse the importance of listening democratically more strongly than the importance of contributing democratically (paired sample t-test: *t* (5155) = 64.62). Furthermore, regarding voice as discussion, students generally reported the classroom climate to be relatively open for discussion (*M = *3.348, *SD* = .672). Regarding voice as influence, 66% of the students indicated to have never participated in any of ‘voice as influence’ related activities (e.g., being a candidate for student council and deciding on how things are done at school).

**Table 1.** Descriptive statistics for individual-level variables.

	*n*	*M*	*SD*	Range
Gender	5167	0.520	.500	0–1
Migration background	5167	0.244	.430	0–1
SES higher	4214	0.542	.498	0–1
Track	5123	0.561	.496	0–1
Voice as discussion	5165	3.348	.672	1–5
Voice as influence	5162	0.339	.473	0–1
Democratic school culture	4949	3.512	.344	1–5
Listening democratically	5156	3.160	.549	1–4
Contributing democratically	5156	2.559	.683	1–4

Bivariate correlations between all variables are presented in Table 2. These correlations show that the two dependent variables in this paper, attitudes concerning contributing democratically and listening democratically are related. Hence, multivariate analyses in which both dependent variables are included in one model is appropriate. Regarding the hypothesised relations, the correlations indicate that, the independent variables, voice as discussion and voice as influence, are related to both dependent variables: contributing and listening democratically. Democratic school culture was not related to the dependent variables. These relations will be examined in the main analyses, while controlling for the nested nature of the data, and while controlling for possible confounders.

**Table 2.** Bivariate correlations between all variables in this study.

	1.	2.	3.	4.	5.	6.	7.	8.	9.	10.	11.
1. Gender											
2. Migration background	.023										
3. SES higher	.006	−.036*									
4. Track	.045**	−.075***	.311***								
5. Voice as discussion	.076***	−.034***	.051***	.084***							
6. Voice as influence	.043**	.026	.031*	.034*	.077***						
7. Voice as discussion – classroom avg.	.003**	−.133***	.127***	.226***	.369***	.046***					
8. Voice as influence – classroom avg.	.015	.044**	.101***	.095***	.049***	.351***	.133***				
9. Democratic school culture	.023	−.093***	−.001	−.153***	.024	.036*	.064***	.103***			
10. Listening democratically	.106***	.011	.064**	.131**	.227**	.071**	.171**	.061**	.001		
11. Contributing democratically	.004	.115***	.106***	.106***	.165***	.157***	.123***	.099***	.016	.429***	

Note. * *p* < .05; ** *p* < .01; *** *p* < .001

### 4.2. Main analyses

The multilevel analyses proceeded stepwise (see Table 3). The first model depicts the baseline differences in democratic voice attitudes and shows that girls valued listening democratically more than boys (*B* = .167). Students with a migration background (*B* = .161) and students with higher educated parents (*B* = .090) valued contributing democratically more than their counterparts. Additionally, students in the general/academic educational tracks valued both contributing and listening democratically more than students in the vocational tracks (respectively, *B* = .126; *B* = .123).

**Table 3.** Coefficients of three-level multivariate regression analyses.

	Model 1		Model 2		Model 3		Model 4		Model 5		Model 6	
	Contributing	Listening	Contributing	Listening	Contributing	Listening	Contributing	Listening	Contributing	Listening	Contributing	Listening
**Student level**												
Gender (ref = boy)	−.004	.167***	−.026	.149**	−.004	.186***	−.024	.152***	−.025	.151***	−.025	.151***
Migration background (ref = native)	.161***	.027	.165***	.032	.179***	.043*	.174***	.041*	.177***	.038*	.177**	.042*
SES (ref = lower)	.090***	.015	.088***	.014	.087***	.014	.087***	.012	.085***	.011	.085***	.012
Voice as discussion			.146***	.158***			.132***	.143***	.132***	.143***	.132***	.145***^a^
Voice as influence			.195***	.049**			.188***	.044*	.188***	.044*	.188***	.045*
**Classroom level**												
Track (ref = lower)	.126***	.133***	.105***	.115***	.088**	.095***	.089**	.096***	.094**	.095***	.094**	.098***
Voice as discussion (average)					.288***	.328***	.154**	.184***	.148**	.182***	.148**	.174***
Voice as influence (perc. active)					.271***	.097+	.085	.054	.077	.053	.076	.06
**School level**												
Democratic school culture									.053	.003	.054	−.002*
**Interaction**												
Democratic school culture * voice as influence												−.003
ICC classroom level	.022	.044	.012	.030	.029	.052	.015	.035	.015	.035	.015	.035
ICC school level	.020	.014	.015	.010	.011	.006	.012	.006	.012	.006	.012	.006

Note. ^a^Random slope. Unstandardised coefficients, all continuous variables grand mean centred. Multivariate analyses: correlations between dependent variables are also modelled. * p < .05; ** p < .01; *** p < .001.

In the second model, the two main independent variables, voice as discussion and voice as influence were added. In support of Hypotheses 1 a and b, the findings in Model 2 show that students who experienced voice as discussion at school have more (a) positive attitudes to contributing democratically and more (b) positive attitudes towards listening democratically (respectively, *B* = .146; *B* = .158). Furthermore, in support of Hypotheses 2 a and b, also students who experienced more voice as influence at school valued both (a) contributing democratically and (b) listening democratically more (in Model 2, respectively, *B* = .195; *B* = .049) more.

These associations were also significant when examined (only) at the classroom level (see Model 3), with the exception of the classroom-level association between voice as influence and listening democratically, which was marginally significant (*p* = .06). Next, Model 4 includes the two independent constructs of voice experiences at the individual and classroom level. In this model, the share of classmates who experienced voice as influence is no longer significantly related to the outcome variables. This indicates that not the experiences of classmates, but particularly students’ personal experiences with voice as influence are positively related to students’ attitudes to voice (contributing: *B* = .188, listening: *B* = .044). Regarding the other independent variable, voice as discussion, both personal and classmates’ experiences are positively related to the extent that students value the two aspects of voice (contributing: *B* = .132 and *B* = .154, listening: *B* = .143 and *B* = .184).

In model 5, democratic school climate is added as a direct effect (not significant and not hypothesised) to subsequently examine whether a democratic school climate strengthens the associations above. To examine whether these cross-level interactions occur, we first examine the classroom and school-level variance of the random slopes for the relations between classroom and school voice and the dependent variables. Only the effect of voice as discussion on listening democratically differs between classrooms (*σ^2^ *= .006, *p* = .041). Hence, only one interaction is entered in Model 6 to examine whether democratic school culture affects the relation (i.e., slope) between voice as discussion and the dependent variable listening democratically. This interaction effect is not significant. This means that there is no support for Hypotheses 3 (a, b, c, d). As such, a democratic school climate, as measured through teachers’ democratic experiences, does not moderate the relation between students’ experiences concerning voice (as discussion and influence) and their attitudes towards voice (regarding democratically contributing and listening).

## 5. Conclusion and discussion

The democratic system is defined by the rule of people through the contribution of their voice, and voice can only serve its purpose if others are willing to listen. As current citizens and future voters and representatives, students’ attitudes towards using their own voice and to listening to the voice of others thus matter. This study addresses this theme from the perspective of schools as practice grounds for citizenship. The objective of this study was to investigate whether students’ voice experiences in school, like engaging in classroom discussions and influencing school policy, are related to students’ democratic attitudes towards voice. More specifically, their attitudes towards valuing the contribution of their own voice and listening to that of others. Additionally, we examined whether this relation was affected by the presence of a more, or less, democratic school culture. To summarise our findings: the results are predominantly consistent with the expectations. Students’ experiences with voice in school were indeed related to their attitudes towards contributing and listening democratically. This underscores the relevance of understanding schools as practice grounds for citizenship (Rinnooy Kan et al. 2021). Contrary to our expectations, this finding is not influenced by the extent to which the school culture is democratic according to teachers.

To examine how a school can function as a practice ground for citizenship, this study first looked at students’ individual experiences with two expressions of voice within the school context: voice as discussion and voice as influence. Voice as discussion primarily concerns students’ voice as part of discussion within classrooms, whereas voice as influence refers to students’ voice as part of decision-making processes related to school policy and/or being a member of the student council. Individual experiences with both these expressions of voice were positively related to students’ attitudes towards contributing and listening democratically. This is in line with previous research that consistently shows the relevance of social participation in school as ‘citizenship learning opportunity’ to help develop students’ citizenship competences (e.g. Hoskins, Janmaat, and Villalba 2012; Kahne and Sporte 2008).

Besides individual experiences, the social context (i.e. behaviour and experiences of classmates) at school matters too. Adding to research on the relationship between citizenship education and democratic attitudes, which primarily focused on individual experiences (and outcomes), this study underscores the social dimension of learning for citizenship within the school context (Bandura and Walters 1977; Wildemeersch 2014). Besides the effect of individual experiences, our results show that a shared classroom experience of voice as discussion positively relates to individual students’ attitudes towards contributing and listening democratically. This illustrates the relevance of a social, more situated learning approach to understand how citizenship learning within the school works. However, classroom-level experiences with voice as influence were not related to students’ attitudes. For future research, it would therefore be relevant to investigate the ways in which social learning can be actively utilised in the context of citizenship education, for example through qualitative research on different modes of participation in the context of intergroup dynamics, to better understand how social processes within the classroom influence the individual student’s experience with voice (cf. Percy-Smith et al. 2020).

Finally, this study went one level higher and examined whether the presence of a democratic school culture strengthened the relation between students’ voice experiences and their outcomes. The findings show that the school culture did not affect the relation between students’ voice experiences at school and their attitudes towards voice. It is possible that school culture simply does not play an important role when it comes to students’ attitudes and that voice experiences promote attitudes towards voice in both more and less democratic school cultures. However, the absence of this moderation may also raise questions about our operationalisation of a democratic school culture, which focuses on teacher experiences. It suggests that taking only teacher experiences in relation to school leadership into account might give too little insight into students’ experience of the so-called ‘shared habitus of participation’ in school (Wood 2014). Moreover, the overview study of Barber, Clark, and Torney-Purta (2021) stresses, on the one hand, the importance of the school climate for students’ civic outcomes, but on the other shows that it affects different students in different ways. To account for these different experiences, more qualitative student-focused research is crucial. In line with the focus of our study, this would, for example, imply gaining more insight into what barriers students experience when it comes to the practice of using their voice even when the opportunities formally do exist. Combining the different perspectives of students and teachers seems crucial, as research implies that teachers and students have differing views on to what extent their school’s culture is democratic and have different understandings of the meaning of a ‘democratic school culture’ (Yavuz Tabak and Karip 2021). Furthermore, it does seem relevant to look at the practices of a broad array of teachers in the school to add to previous research that mainly focusses on civics and social studies teachers, to acknowledge that each classroom holds the potential of being characterised by a democratic culture (cf. Barber, Clark, and Torney-Purta 2021).

Additionally, this study invites reflection on the practice of ‘voice as influence’ in schools. Voice as influence is considered one of the most concrete ways in which students can practice (for) democracy. However, in our sample only a very limited number of students had any experience in this realm (24,7%). This limited access to the experience of voice as influence is in line with what other research shows (cf. Taylor and Robinson 2009). Additionally, previous studies indicate that the actual influence students have even when they practice voice in this realm, for example in a student council, is limited and that students are primarily invited in a tokenistic way (cf. Hall 2017; Charteris and Smardon 2019). In the light of these previous studies, the implications in this study are twofold. On the one hand, this study shows that experiencing voice, even when not directly related to influence on school policy, still positively relates to important democratic outcomes. On the other hand, the findings point towards missed opportunities, as so few students directly experience ‘voice as influence’, whereas it seems particularly strongly related to students’ attitudes towards both listening democratically and contributing democratically. As such, these results can be considered as an invitation for school leaders and teachers to facilitate a broader range of opportunities for all students to practice voice as influence (cf. Evans 2006; Leenders, Veugelers, and De Kat 2008; Patterson, Doppen, and Misco 2012).

This study extends previous research on democratic attitudes of adolescents in two important ways. First, research on citizenship and education includes a broad array of practices ranging from a formal civics curriculum to elements of the school climate. The same holds true for outcomes considered ‘democratic (or civic, or political) attitudes’ ranging from political interest to the belief that one should contribute one’s own voice. More insight into what practices relate to what specific outcomes can help educators to understand better what they can offer students to fulfil their specific citizenship education goals (cf. Dassonneville et al. 2012). In this study, we therefore connected voice-related practices to voice-related outcomes, and we looked at three different mechanisms potentially underlying the relationship. Secondly, the current study examined both contributing and listening as two sides of democratic voice. Even though listening is just as essential for discussions as contributing, listening is rarely considered a democratic practice, and that also holds true in the educational context (Dobson 2014). This is underlined by the fact that the sparse research considering adolescents’ attitudes towards voice primarily focusses on their intention to use their own, by voting in a future election (e.g. Cohen and Chaffee 2013). In a recent aberrant study, empathetic listening is operationalised as a civic skill and crucial part of democratic education (Andolina and Conklin 2021). This perspective implies that both in the context of citizenship education and citizenship education research, the practice of listening deserves to get more attention.

In terms of limitations, it is important to consider the option that the causality is reversed and that students with more positive attitudes towards contributing and listening democratically pursue and find more opportunities to practice their voice, especially for voice as discussion. This could also be a mutually reinforcing relationship. In terms of practical implications, this does not affect the importance of reflecting on ways to offer students as much opportunity as possible to practice voice as discussion and voice as influence. Furthermore, there might be additional instances where voice (and listening) is practiced that we have not taken into account but are relevant, such as less supervised instances between peers. Finally, as our results indicated no moderation by democratic school culture, an improved measurement where student experiences are considered alongside that of teachers might give us more insight in the role of democratic school culture in the context of schools as practice grounds for citizenship.

To conclude, using the framework of schools as practice grounds enabled us to illuminate the importance for all students to have different opportunities to practice voice in a school context, as individuals but also as members of a classroom community. Moreover, this study draws further attention to the fact that a democratic mindset includes listening, which may just be a civic skill our democracies in distress are longing for.
